# TRAF6 Promoted Tumor Glycolysis in Non-Small-Cell Lung Cancer by Activating the Akt-HIF*α* Pathway

**DOI:** 10.1155/2021/3431245

**Published:** 2021-08-06

**Authors:** Lihua Feng, Shuitu Feng, Zhihua Nie, Yan Deng, Yingmei Xuan, Xiaoping Chen, Yaqun Lu, Lina Liang, Yide Chen

**Affiliations:** Haicang Hospital, No. 89 Haiyu Road, Haicang District, Xiamen, China

## Abstract

TRAF6 has been reported to be associated with poor prognosis in non-small-cell lung cancer (NSCLC). However, its precise role in tumor development has not been elaborated. In the present study, the function and the mechanism by which TRAF6 contributes to development were intensively investigated. TRAF6 was found to be overexpressed in primary NSCLC tumor tissue and all tested cell lines. Knockdown of TRAF6 with shRNA substantially attenuated NSCLC cell proliferation and anchorage-independent growth. Moreover, tumor glycolysis, such as glucose consumption and lactate production, also significantly impaired. In TRAF6-deficient cells, hexokinase-2 expression was significantly reduced, which was caused by the decrease of HIF-1*α* transcriptional activity. Further investigations demonstrated that TRAF6 played an important role in the regulation of Akt activation, and exogenous overexpression of constitutively activated Akt substantially rescued glycolysis suppression in TRAF6 knockdown cells. The results of the xenograft model confirmed that downregulation of TRAF6 in NSCLC tumor cells dramatically restrained tumor growth in vivo. Taken together, our studies revealed the mechanism by which TRAF6 exerts its role in NSCLC development and suggested TRAF6 maybe was a promising candidate target for lung cancer prevention and therapy.

## 1. Introduction

When oxygen is available, the energy supply in normal cells is powered by the oxidative phosphorylation systems (OXPHOS) in mitochondria. However, with no use of OXPHOS, glycolysis is utilized by tumor cells to consume more glucose and produce large amounts of lactic acid, even in the presence of oxygen, a phenomenon known as aerobic glycolysis, or the Warburg effect [1]. Although tumor glycolysis is less efficient than OXPHOS in terms of ATP production, the shift from OXPHOS to tumor glycolysis is critical for tumor cells to satisfy its rapid proliferation and is considered to be an important hallmark of cancer cells [2]. Owing to the rapid growth, tumor cells are in a state of long-term hypoxia. The active glycolysis can strengthen the tolerance to hypoxia, avoiding cell apoptosis induced by OXPHOS inhibition. Moreover, the intermediate metabolites generated in glycolysis provide sufficient raw materials for tumor anabolism and promote the synthesis of fatty acids and nucleic acids [3]. The lactate produced by tumor glycolysis creates an acidic environment, which can break down the extracellular matrix and thus facilitate the invasion and migration of cancer cells [4]. Recent studies have shown that lactate can also regulate the function of dendritic cells in the tumor microenvironment and affect the immune responses [5, 6].

TRAF6 belongs to the tumor necrosis factor receptor-associated factor (TRAF) family, which was originally discovered as signal transduction molecule in tumor necrosis factor receptor- (TNFR-) mediated signaling pathway [7]. Like other members of the family, TRAF6 has a highly conserved TRAF domain at the C-terminal, which is important for TRAF6 to exert its role as a signaling adaptor and is involved in the association with upstream receptors and downstream proteins [8]. In the N-terminal, there is a conserved RING finger domain, which is necessary for TRAF6 to function as an E3 ubiquitination ligase. Upon binding with E2 conjugating enzyme, such as Ubc13/Uev1A, TRAF6 can mediate the Lys-48 polyubiquitination of the target protein and give rise to the activation of downstream signaling pathways [9]. Except for TNFR signaling, increasing evidence demonstrated that TRAF6 played a critical role in other immune signaling, including Toll-like receptors (TLRs), RIG-1-like receptors, IL-1 receptor (IL-1R), and IL-17R [10–13]. Several studies demonstrated that TRAF6 was abnormally expressed and activated in tumor tissue, such as esophageal squamous cell carcinoma (ESCC), colon cancer, and pancreatic cancer [14–16]. In lung cancer, TRAF6 was found to be overexpressed and the overexpression was closely associated with the clinical TNM stage, tumor size, and lymph node metastasis [17]. So far, the underlying mechanisms by which TRAF6 was upregulated in tumor tissue are largely unknown. In lung cancer, TRAF6 locus amplification was found to occur frequently, and TRAF6 plays an important role in connecting RAS and NF-*κ*B signaling pathways [18]. In lung adenocarcinoma development and progression, TRAF6 was proved to be involved in the regulation of tumor cell proliferation, invasion, metastasis, and apoptosis resistance [19, 20]. However, the role of TRAF6 played in tumor glycolysis was largely unknown. In the present study, we aimed to elucidate the effects of TRAF6 on tumor glycolysis as well as the underlying mechanisms.

## 2. Material and Methods

### 2.1. Cell Line and Reagent

The normal human bronchial epithelioid cell (HBE) and the non-small-cell lung cancer cell lines including NCI-H1650, NCI-H1299, A549, NCI-H460, HCC4006, NCI-H1975, and NCI-H358 were obtained from ATCC and cultured with recommended culture medium. The primary antibodies for western blotting including anti-TRAF6 (#8028), hexokinase-1 (#2024), hexokinase-2 (#2867), GLUT1 (#12939), PKM2 (#4053), LDHA (#35 82), VDAC-1 (#4661), phosphor-Akt (#4060), Akt (#8596), phosphor-S6 (#4858), and ubiquitin (#58395) as well as the secondary anti-rabbit IgG HRP (#7074) were products of Cell Signaling Technology Inc. (Danvers, MA). *β*-Actin (A5316) was obtained from Sigma-Aldrich. In immunohistochemistry staining, the primary antibodies against hexokinase-2 (ab227198) and Ki67 (ab15580) were products of Abcam. TRAF6 shRNA#1 (TRCN0000007350) and shRNA#2 (TRCN0000007351) were purchased from the Sigma Mission shRNA library. The constitutively active Akt (CA-Akt) plasmid (Cat. #10841) and pLKO.1 GFP shRNA (Cat. #30323) were purchased from Addgene (Cambridge, MA, USA). Recombinant human insulin-like growth factor 1 (IGF-1) was a product of R&D (Cat. 291-G1-200). Lipofectamine 2000 was obtained from Invitrogen (Carlsbad, CA).

### 2.2. Generation of shRNA Knockdown Cells

Firstly, the plasmid pLKO.1-sh-TRAF6 and helping plasmids psPAX2 (#12260, Addgene) and PMD2.G (#12259, Addgene) were transfected into HEK-293T cells. Eight hours posttransfection, the cells were cultured with fresh medium. After 48 hours, the supernatant with lentivirus was harvested, and the cell debris was removed with a 0.45 *μ*m filter. The NSCLC cells were infected with the supernatant containing TRAF6-shRNA lentivirus to generate TRAF6 knockdown cells. The cells were then selected with 5 *μ*g/ml puromycin for about 7-10 days, and the knockdown efficiency was validated with western blotting.

### 2.3. Cell Proliferation Assay

In cell proliferation assay, 3000 cells/well were seeded into a 96-well plate and at different time points (0, 24, 48, and 72 hours after seeding), Cell Titer-Glo (Promega) reagent was added into the plate, and the luminescence was detected following the manufacturer's protocol.

### 2.4. Colony Formation Assay

Firstly, 1 ml Eagle's basal medium supplemented with 0.6% agar and 10% FBS was loaded into a 6-well plate as the agar base. After the agar solidified, appropriate cells (6 × 10^3^/well) were seeded into the plate with 0.3% Basal Medium Eagle agar containing 10% FBS. The plates were cultured for 2 weeks, and the number of colonies was counted.

### 2.5. Tumor Glycolysis Measurement

Glucose consumption and lactate secretion were assessed with the Glucose-Glo™ (Cat. J6021, Promega) and Lactate-Glo™ (Cat. J5021, Promega) assay kit, respectively. Briefly, NSCLC cells (2 × 10^4^/well) were seeded in a 96-well plate. After the cells attached to the plate, the cells were washed with culture medium twice and renewed with fresh culture medium. Eight hours later, the concentration of glucose and lactate in the medium was determined according to the supplier's instructions. The rate of glucose consumption and lactate production was normalized against the protein concentration.

### 2.6. Mitochondrial Isolation

Cell pellets were harvested by centrifugation at 500 g for 5 mins, and the mitochondrial fraction was isolated with the Mitochondria Isolation Kit (Cat. 89874, Thermo Fisher Scientific) by following the supplier's instructions.

### 2.7. Western Blotting

Tumor cells were lysed with the RIPA lysis buffer supplemented with the cocktail inhibitor, and the lysates were centrifuged; then the supernatant was collected, and the concentration was determined with the bicinchoninic acid method (Cat. 23227, Thermo Fisher Scientific, Asheville, NC, USA). An equal amount of protein per lane was loaded and resolved by the SDS-PAGE, then electrophonically transferred to the polyvinylidene difluoride membrane. Blocking unspecific binding with nonfat milk, the membrane was incubated with a specific primary antibody. After the hybridization with the horseradish peroxidase- (HRP-) conjugated secondary antibody, the band on the membrane was visualized with the chemiluminescence reagent.

### 2.8. RT-PCR

RNA extraction was performed with TRIzol™ reagent (Cat. 15596026, Thermo Fisher Scientific) by following the standard protocols, and the complementary cDNA was prepared with the SuperScript™ III Reverse Transcriptase (Cat. 18080093, Thermo Fisher Scientific). The following primers were used in reactions: hexokinase-2—sense 5′-ATTGTCCAGTGCATCGCGGA-3′, antisense 5′-AGGTCAAACTCCTCTCGCCG-3′; GAPDH—sense 5′-ACCCAGAAGACTGTGGATGG-3′, antisense 5′-AGTAGAGGCAGGGATGATGTT-3′. The reaction was performed with the QuantiTest SYBR Green PCR kit (Cat. 204143, QIAGEN) on ABI7500Fast, cycling between 95 and 60 for 50 cycles. The abundance of mRNA was quantified and normalized against corresponding GAPDH mRNA loaded.

### 2.9. HIF-1*α* Reporter Gene Assay

Briefly, 4 × 10^4^ cells per well were plated in 24-well plates and cultured with medium without antibiotics. For transfection, the HRE-fluc plasmids and SV40-rluc plasmids were diluted with Opti-MEM (Gibco) and then mixed with the Lipofectamine 2000 and incubated at room temperature for 10 mins. Before cell transfection, the cell culture medium was renewed, and the mixture was added. Twenty-four hours later, the culture medium was replaced with fresh medium, and the cells were exposed to hypoxia for 8 hours. The activities of luciferase were evaluated with Dual-Luciferase Reporter Assay System (Cat. E1960, Promega, Fitchburg, WI, USA) by following protocols. The ratio of Firefly/Renilla luciferase was normalized with the protein concentration.

### 2.10. In Vivo Experiment

BALB/ca (eight weeks old) nude mice were fed under specific pathogen-free (SPF) conditions, and the experiment was carried out with the approval of the Animal Care & Use Committee. After the adaptation period, 0.1 ml cell suspension (5 × 10^6^ cells) mixed with the same volume of Matrigel was s.c. injected into the right flank of mice. When the tumor formed, the tumors were measured with micro calipers twice per week, and the volume was calculated as length × width^2^/2. At the end of the experiment, the mice were sacrificed, and the tumors were weighed and photographed.

### 2.11. Immunohistochemistry Staining

The paraffin-embedded tissues were dewaxed with xylene and then hydrated with a gradient of 100% to 75% alcohol. After the antigen retrieval with boiling sodium citrate buffer (10 mmol/l, pH 6.0), the tissue was treated with 3% H_2_O_2_ for 10 minutes. Incubation with the serum from the host of the secondary antibody to block nonspecific binding sites, the tissue was incubated with primary antibodies against HK2 (1 : 200) or Ki67 (1 : 250), respectively, in a humidified chamber at 4°C overnight. After washing, the sections were hybridized with the biotinylated secondary antibody and followed by incubation with HPR-conjugated streptavidin using the Vectastain Elite ABC kit (Vector Laboratories, Inc.). The peroxidase activity was visualized with 3,3-diaminobenzidine (DAB) solution. After washing with distilled water, the slides were counterstained with hematoxylin, dehydrated, and mounted.

### 2.12. Statistical Analysis

The statistical analysis was performed with SPSS software (version 13.0), and the method of a two-tailed Student's *t*-test was employed. *p* < 0.05 was considered to be a significant statistical difference.

## 3. Results

### 3.1. TRAF6 Is Overexpressed in NSCLC and Important for Tumor Cell Proliferation

**Figure 1:**
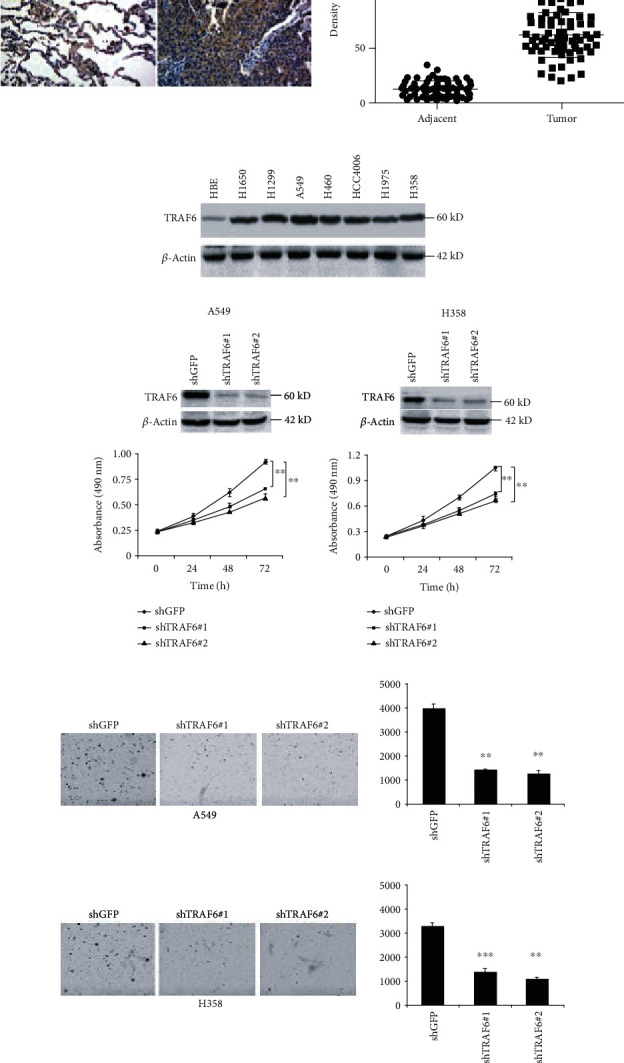
TRAF6 was overexpressed in NSCLC and mediates cell proliferation. (a) The expression of TRAF6 in paired NSCLC tissue microarray. Left: the representative images; right: quantitative expression of TRAF6 in 75 paired NSCLC tissue (*p* < 0.001). (b) The expression of TRAF6 in seven NSCLC cell lines. (c) TRAF6 knockdown inhibited tumor cell proliferation. Upper: TRAF6 expression after shRNA transfection was examined by western blotting; below: cell proliferation after TRAF6 knockdown was examined as described. (d) TRAF6 shRNA suppressed colony formation of NSCLC. Left: representative images; right: the number of clones was quantified. ^∗∗^*p* < 0.01 and ^∗∗∗^*p* < 0.001 vs. the sh-GFP group.

Firstly, we investigated the expression of TRAF6 in 75 paired NSCLC tissue. As shown in Figure 1(a), in comparison with the adjacent normal tissue, TRAF6 expression in NSCLC tumor tissue is substantially higher. Moreover, the results of western blotting demonstrated slight TRAF6 expression were detected in normal bronchial epithelial HBE cells, while in other tested seven NSCLC cell lines, the expression of TRAF6 was significantly elevated. To study the biological function of TRAF6, specific shRNAs were employed to silence TRAF6 expression; then the effect of shRNA on cell proliferative ability was measured. In TRAF6-deficient cells, the cell proliferation was dramatically affected, with a decrease of about 50%. Meanwhile, we also examined the effect of TRAF6 on cell colony formation. As the results have shown in Figure 1(d), after the knockdown of TRAF6, the number of tumor clones formed in soft agar was significantly reduced, suggesting that TRAF6 has an important role in tumor development.

### 3.2. TRAF6 Played an Important Role in Tumor Glycolysis Regulation

**Figure 2:**
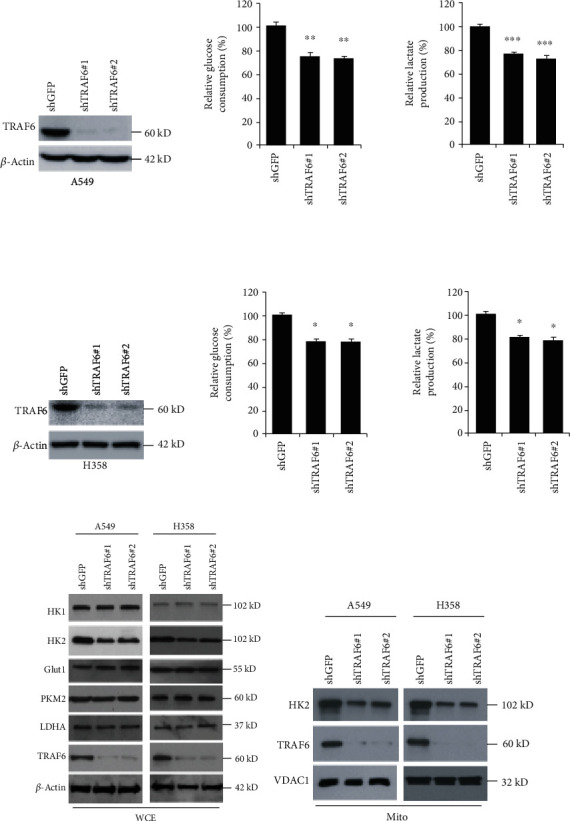
TRAF6 mediated tumor glycolysis in NSCLC. (a) TRAF6 shRNAs inhibited tumor glycolysis. After the transfection of TRAF6-shRNA, glucose consumption and lactate production were determined as described. (b) TRAF6 shRNAs suppressed hexokinase-2 expression. In TRAF6-deficient cells, the expression of pivotal proteins in the glucose metabolism pathway was examined. (c) TRAF6 shRNA inhibited hexokinase-2 expression in mitochondria. The mitochondria fraction was extracted, and hexokinase-2 was analyzed by western blotting. ^∗^*p* < 0.05, ^∗∗^*p* < 0.01, and ^∗∗∗^*p* < 0.001 vs. the sh-GFP group.

With the knockdown of TRAF6 in NSCLC, we examined the effect of shTRAF6 on tumor glycolysis by measuring the glucose uptake and lactate production. As the results have shown in Figure 2(a), the glucose consumption was decreased about 30% in the absence of TRAF6. Consistent with the reduction of glucose uptake, the amount of lactate generated by TRAF6-deficient NSCLC cells was also substantially declined. To explore the mechanism by which tumor glycolysis was inhibited, the effects of TRAF6 shRNA on the glucose metabolism pathway were investigated. As demonstrated, TRAF6 knockdown resulted in a remarked suppression of hexokinase-2 while it had no obvious effect on other kinases or transporters (Figure 2(b)). Given the fact that hexokinase-2 is mainly located in mitochondria to exert its biological functions, the mitochondrial fractions were extracted, and hexokinase-2 was measured. As shown in Figure 2(c), in contrast with the mock group, hexokinase-2 in mitochondria was significantly decreased accordingly.

### 3.3. HIF-1*α* Was Involved in TRAF6-Mediated Hexokinase-2 Suppression

**Figure 3:**
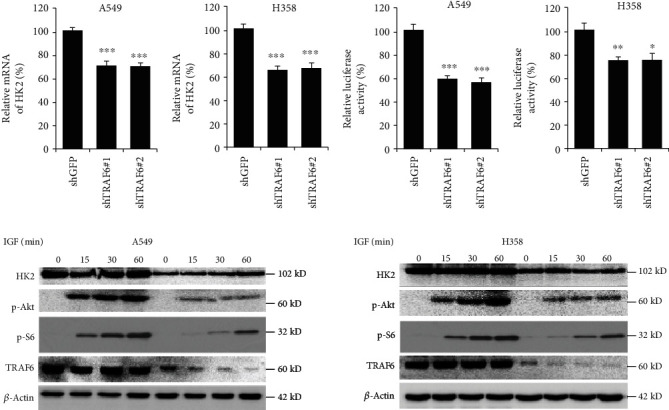
Akt-HIF-1*α* axis was involved in TRAF6-mediated tumor glycolysis. (a) TRAF6 shRNAs suppress hexokinase-2 transcription. Hexokinase-2 mRNA levels were quantified by RT-PCR as described. (b) The transcriptional activities of HIF-1*α* were decreased after TRAF6 knockdown. HIF-1*α* reporter gene was transfected into NSCLC cells, and the effect of TRAF6 shRNA on HIF-1*α* transcriptional activities was evaluated. (c) TRAF6 shRNA inhibited Akt activation in NSCLC. After TRAF6 shRNA transfection, the phosphorylation of Akt, S6, and hexokinase-2 expression was examined. ^∗^*p* < 0.05, ^∗∗^*p* < 0.01, and ^∗∗∗^*p* < 0.001 vs. the sh-GFP group.

To explore the underlying mechanism, we adopted real-time PCR to study the effect of sh-TRAF6 on hexokinase-2 mRNA. As shown in Figure 3(a), in TRAF6 knockdown cells, the amount of hexokinase-2 mRNA was decreased, suggesting the inhibition of transcription contributed to the decrease of hexokinase-2 by sh-TRAF6. Transcriptional factor HIF-1*α* was reported to be important for hexokinase-2 transcriptional regulation. To validate the role of HIF-1*α*, we constructed the HIF-1*α* reporter gene and tested the effect on HIF-1*α*. In contrast with the control cells, the activities of luciferase in TRAF6 silenced cells were significantly decreased (Figure 3(b)), suggesting the transcriptional activity of HIF-1*α* was substantially inhibited after TRAF6 knockdown in NSCLC cells. Furthermore, after the stimulation of insulin-like growth factor-1 (IGF-1), in TRAF6-deficient cells, the activation of Akt was dramatically suppressed. With the inhibition of Akt, the activity of its downstream signal factor, such as S6, was also substantially downregulated, and hexokinase-2 expression was decreased accordingly, suggesting the AKT-HIF-1*α* axis was involved in the regulation of hexokinase-2 by TRAF6 (Figure 3(c)).

### 3.4. TRAF6 Mediated Tumor Glycolysis by Akt Ubiquitination

**Figure 4:**
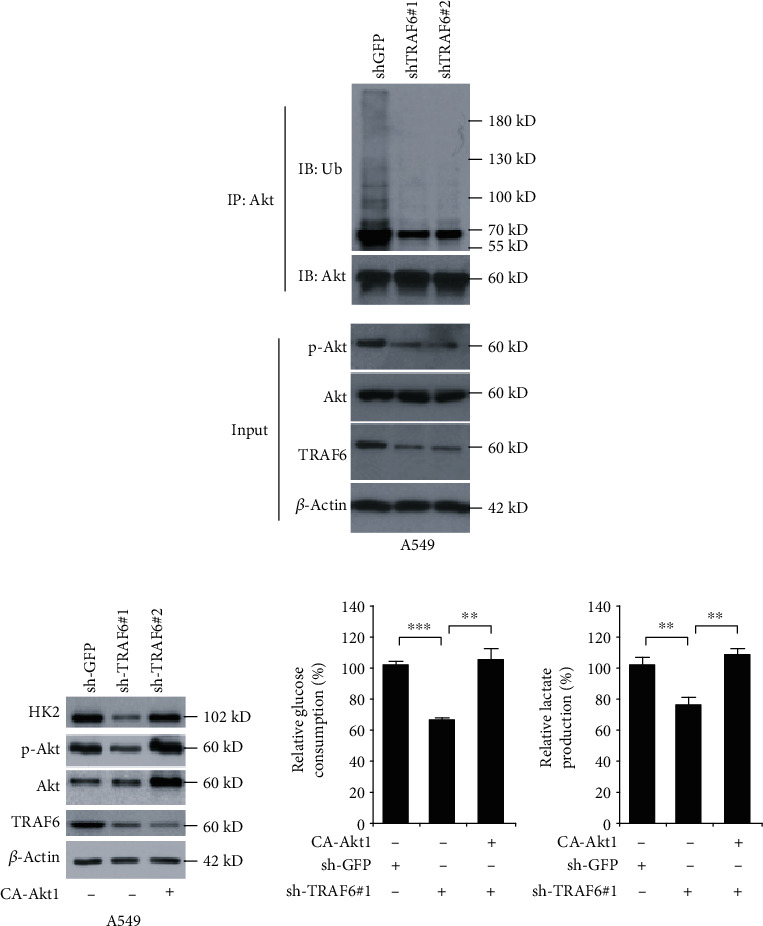
Akt played a critical role in TRAF6-mediated tumor glycolysis. (a) TRAF6 mediated Akt ubiquitination and activation in A549 cell. In TRAF6 knockdown A549 cells, the ubiquitination status of Akt was analyzed by immunoprecipitation as described. (b) The exogenous introduction of CA-Akt1 attenuated glycolysis suppression. In TRAF6 knockdown A549 cells, CA-Akt1 was introduced, and the effects on hexokinase-2 and tumor glycolysis were studied. ^∗∗^*p* < 0.01 and ^∗∗∗^*p* < 0.001; Student's *t*-test.

As previously reported, ubiquitination is critical for Akt membrane translocation and activation, so the ubiquitination status of Akt was measured by immunoprecipitation. As shown in Figure 4(a), the ubiquitination levels of Akt were relatively high in the untreated cells, whereas in TRAF6 knockdown cells, Akt ubiquitination was substantially decreased. To further validate the importance of Akt in TRAF6-mediated tumor glycolysis, constitutively activated Akt was introduced into TRAF6-deficient cells. As the results demonstrated in Figure 4(b), after the CA-Akt1 transfection in TRAF6-deficient cells, the phosphorylation of Akt was significantly increased, and hexokinase-2 expression was substantially elevated. Accordingly, the glycolytic capabilities, including glucose consumption and lactate production, were dramatically recovered.

### 3.5. TRAF6 Shrna Inhibited Tumor Growth *In Vivo*

**Figure 5:**
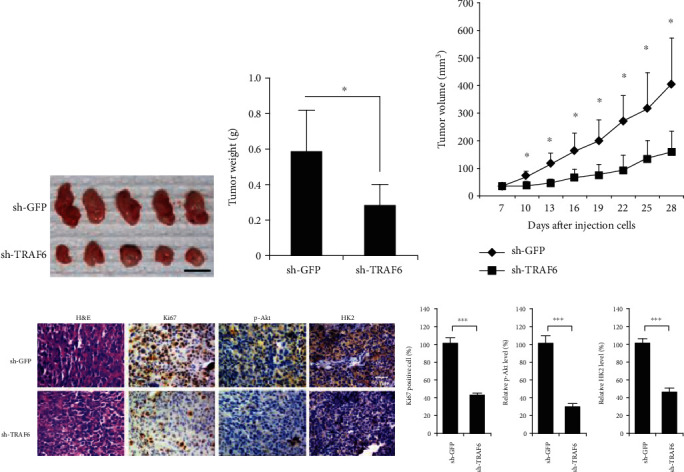
TRAF6 shRNA suppresses tumor growth in vivo. (a) The images of tumor in nude mice; scale bar, 1 cm. (b) The weight of tumors. (c) The tumor growth curve. (d) The immunohistochemical staining of Ki67, TRAF6, and hexokinase-2 in tumor tissue. ^∗^*p* < 0.05 and ^∗∗∗^*p* < 0.001 vs. the sh-GFP group.

To examine the effect of TRAF6 shRNA on tumor growth in vivo, A549 cells with or without TRAF6 shRNA were injected into nude mice, and the tumorigenicity was observed. As shown in Figures 5(a)–5(c), in comparison with the control, the growth rate of the TRAF6 shRNA group was substantially attenuated. Twenty-eight days after cell inoculation, the tumor volume in the control group was over 400 mm^3^, while in the TRAF6 shRNA group it was about 150 mm^3^ (*p* < 0.05), and the tumor weight is also statistically different (0.58 g vs. 0.23 g, *p* < 0.05). Immunohistochemistry results demonstrated that, in the tumor tissue of the TRAF6 shRNA group, Akt phosphorylation was substantially decreased, and the expression of hexokinase-2 was also dramatically reduced, which was in accordance with the in vitro results. Moreover, Ki67, an important biomarker of cell proliferative potential, was also substantially impaired in TRAF6-deficient tumor tissue (Figure 5(d)).

## 4. Discussion

In tumor development, to meet the energy requirement of rapid proliferation, it is necessary for tumor cells to reprogram cellular metabolism. A series of complex factors, for instance, the change of tumor microenvironment, the activation of oncogenes, and the inactivation of tumor suppressor genes, contributes to the transition from oxidative phosphorylation to tumor glycolysis [21–24]. As a key factor in the various critical signaling pathway, TRAF6 overexpression was conceived to be closely correlated with tumor development and poor clinical prognosis [25–27]. In the present study, we demonstrated that by mediating Akt ubiquitination and activation, regulating HIF-1*α* transcriptional activities, and hexokinase-2 expression, TRAF6 played an important role in the regulation of tumor glycolysis. In TRAF6-deficient NSCLC cells, the glycolytic levels were substantially decreased. With the inhibition of tumor glycolysis, the proliferative potentials and colony formation abilities were dramatically suppressed.

As an important kinase, Akt is at the crossroads of different signaling pathways and mediates tumor cell proliferation, survival, metabolism angiogenesis, and metastasis [28]. Owing to its importance, the activity of Akt is strictly regulated. It is well recognized that the phosphorylation of Akt is critical for its activation. Upon upstream stimulus, the phosphorylation at Ser308 by PDK1 activates Akt, and sequential phosphorylation at Thr473 makes its full activation [29]. Except for phosphorylation, recent studies demonstrated the ubiquitination was also involved in Akt regulation, especially the k63-linked ubiquitination promoted Akt translocation to the membrane [30]. However, the E3 ligase that mediates Akt ubiquitination is cell type or stimulus dependent. The Skp2-SCF E3 ligase was identified to be engaged in EGF-stimulated Akt ubiquitination in breast cancer cells [31]. In fibroblasts, IGF-1 or insulin-induced Akt ubiquitination was mainly dependent on NEDD4-1 [32]. In TRAF6 knockdown NSCLC cells, the ubiquitination level of Akt and IGF-1-induced Akt phosphorylation was significantly decreased, suggesting TRAF6 was the E3 ligase responsible for Akt ubiquitination in NSCLC. Exogenous overexpression of activated Akt substantially rescinded the glycolysis inhibition caused by TRAF6 knockdown, demonstrating Akt had a critical role for TRAF6 to exert its effect on tumor glycolysis.

Hexokinase-2 is indispensable for the conversion of glucose to glucose-6-phosphate, which is the first irreversible step in the glycolytic pathway [33]. By positioning itself in the outer membrane of the mitochondria, HK-2 not only can easily get access to newly synthesized ATP but also can avoid the inhibition of newly produced glucose-6-phosphate [34]. With the knockdown of TRAF6 in NSCLC cells, no obvious changes of hexokinase-1, GLUT1, LDH, and PKM2 were observed. In contrast, the expression of hexokinase-2, both the total and the mitochondria fraction, was substantially decreased, implying hexokinase-2 was involved in TRAF6-mediated tumor glycolysis.

As a pivotal protein in glucose metabolism, hexokinase-2 is regulated at different levels. In gene transcription, different transcriptional factors, including STAT3, p53, and c-myc, were reported to be involved in the mediation of hexokinase-2 [35, 36]. In addition, various posttranslational modifications such as phosphorylation and ubiquitination were implicated [37, 38]. In the absence of TRAF6, hexokinase-2 mRNA was significantly decreased, implying hexokinase-2 decrease may be attributed to the inhibition of its transcription. In our study, we demonstrated that the transcriptional activity of HIF-1, a well-known factor, was dramatically impaired in TRAF6 knockdown cells. HIF-1 consisted of two subunits, HIF-1*α*, which is expressed in an oxygen-dependent manner, and HIF-1*β*, which is constitutively expressed [39]. Generally, under nonhypoxia conditions, the HIF-1*α* subunit is modified by hydroxylation and sequential degradation, resulting in lower activity. In contrast, in hypoxia, the stability of HIF-1*α* was significantly enhanced, and the transcriptional activity of HIF-1 was substantially increased. Except for the oxygen-dependent regulation mechanism, some studies also revealed the activity of HIF-1 was mediated in an oxygen-independent way. In cancer cells, the hyperactivation of growth factor-induced signaling pathways or loss of tumor suppressor genes was suggested to be implicated as HIF-1 regulators [40–42]. As the crossroads of multiple signaling pathways, the activity of Akt had important effects on HIF-1 transcriptional functions. p70S6K1, a kinase downstream of Akt, is involved in the regulation of HIF-1*α*. The activation of the Akt/p70S6K1 pathway led to the increase of translation of HIF-1*α* and HIF-1 target genes in various cellular contexts [43–45]. With the silence of TRAF6, the activity of Akt was decreased, which resulted in the impairment of HIF-1 transcriptional activity and reduction of hexokinase-2.

Briefly, in the present study, we investigated the role of TRAF6 played in tumor glycolysis and the underlying mechanisms. By mediating Akt ubiquitination, TRAF6 promoted Akt activation and enhanced HIF-1 mediated transcription of hexokinase-2, giving rise to the increase of tumor glycolysis in NSCLC. Our studies demonstrated that TRAF6 had an important role in the regulation of tumor glycolysis and was a promising therapeutic target for tumor management.

## Data Availability

The data used to support the findings of this study are included in the article.
